# Acute and Chronic Pancreatitis in Mitochondrial Disease: A Systematic Review

**DOI:** 10.1002/jmd2.70125

**Published:** 2026-09-07

**Authors:** Olivia Hahl, Mika H. Martikainen

**Affiliations:** ^1^ Research Unit of Clinical Medicine, Neurology University of Oulu Oulu Finland; ^2^ Neurocenter and Medical Research Center Oulu University Hospital Oulu Finland

**Keywords:** m.3243A>G, mitochondrial disease, mitochondrial DNA, pancreatitis, systematic review

## Abstract

Mitochondrial disease is a common inherited multisystem neurometabolic disorder. Pancreatic dysfunction is a recognised manifestation, most frequently presenting as mitochondrial diabetes. Although pancreatitis cases have been reported in association with mitochondrial disease, acute and chronic pancreatitis in this context remain poorly characterised. Following the PRISMA framework, we performed a systematic literature review to identify all published cases in which acute or chronic pancreatitis occurred in individuals with a genetically confirmed mitochondrial disease. Literature search yielded 604 publications, of which 19 fit the inclusion criteria. During revision two additional publications were identified, one of which fit the inclusion criteria. These 20 reports described 24 individuals with mitochondrial disease and documented history of acute or chronic pancreatitis. Mean age at first recorded pancreatitis was 13 years, with median age 10 years (range 3 months to 53 years). Pancreatitis was recurrent or chronic in 63% of cases. The most reported presenting symptoms were abdominal pain (38%) and vomiting (33%). None had established pancreatitis risk factors such as gallstones or alcohol misuse. In those who underwent imaging, no structural abnormalities of the pancreas or biliary tree were identified. Most frequent genetic aetiologies were large‐scale mitochondrial DNA (mtDNA) deletions and the m.3243A>G mtDNA variant (29% each). Six patients (25%) died shortly after hospital admission with pancreatitis. Pancreatitis associated with mitochondrial disease often presents in childhood and is frequently recurrent or chronic. Although uncommon, it represents a clinically significant and potentially life‐threatening complication that warrants increased awareness.

## Synopsis

Pancreatitis in patients with a genetically confirmed mitochondrial disease presents typically in paediatric age, without established risk factors and the mortality rate is significant.

## 1 Introduction

Mitochondria are essential organelles present in all eukaryotic cells. Generating energy in the form of adenosine phosphate (ATP) via oxidative phosphorylation is the key function of mitochondria [1]. Consequently, defects in mitochondrial function and thus cellular oxidative energy production affect basically all organs and organ systems, manifesting most commonly in tissues with high energy demand, such as the brain, heart, endocrine pancreas and skeletal muscle [2]. Mitochondrial function is controlled by both the mitochondrial and nuclear genomes, therefore pathological variants in either nuclear DNA or mitochondrial DNA (mtDNA) can contribute to the development of mitochondrial dysfunction [1]. Mitochondrial diseases are a clinically heterogeneous group of multi‐system disorders that are characterised by genetic defects in mitochondrial oxidative phosphorylation [3]. Genetic testing to detect recognised pathogenic variants is the gold standard for diagnosis of mitochondrial diseases. Whilst specific syndromes within the disease group are rare, the overall prevalence of mitochondrial diseases is approximately 1 in 5000 people [4].

The pancreas is a secretory organ located in the retroperitoneum. It contains an exocrine and an endocrine component. The former includes producing, storing and secreting digestive enzymes and the latter includes producing various hormones that regulate blood glucose levels, as well as splanchnic secretion. The endocrine pancreas is composed of five different islet cells, of which approximately 75% are insulin‐producing beta cells [5].

Acute pancreatitis is one of the most common gastrointestinal conditions to result in hospital admission, with gallstones and excessive alcohol use being the leading causes. Other aetiologies include hypertriglyceridemia, hypercalcemia, iatrogenic causes including endoscopic retrograde cholangiopancreatography and endoscopic ultrasonography and medications such as azathioprine, valproate, hydrochlorothiazide and tetracycline. Abdominal pain is the most commonly presenting symptom, often accompanied by nausea, vomiting and fever [6]. As per the Revised Atlanta Classification, to determine a diagnosis of acute pancreatitis, a patient must present with 2 of the following: abdominal pain, an elevated serum amylase and/or lipase greater than 3 times the upper limit of the reference value and cross‐sectional imaging findings consistent with acute pancreatitis [7]. Patients with a history of acute pancreatitis are more susceptible to recurrent episodes because of behavioural or genetic factors. Repeated pancreatic parenchymal injury and inflammation caused by recurrent acute pancreatitis lead to fibrosis and ductal obstruction. This in turn causes pancreatic atrophy and necrosis, leading to chronic pancreatitis. However, in some patients, signs of chronic pancreatitis such as calcification and ductal changes are already present at first admission. The cause of this is unknown, although it may be related to the degree of inflammation or individual patient pain threshold [8].

Mitochondria play a big part in insulin synthesis, as well as sensing and regulating glucose levels. Endocrine pancreas dysfunction manifesting as diabetes mellitus is a well‐described characterisation of mitochondrial diseases [9]. Multiple different mtDNA variants have been correlated with mitochondrial diabetes, although m.3243A>G is the most common [10]. The pathophysiology behind mitochondrial diabetes is apparently a combination of impaired pancreatic insulin secretion due to gradual beta cell failure and insulin resistance in skeletal muscle [11]. Exocrine pancreatic insufficiency has been described in relation to mtDNA deletions presenting as Pearson syndrome (OMIM: 557000), often characterised by refractory sideroblastic anaemia, thrombocytopenia, neutropenia, pancreatic insufficiency and hepatic dysfunction [12].

Acute and chronic pancreatitis have been occasionally described in relation to mitochondrial diseases. The reports have mainly consisted of single patient cases, and a comprehensive summary of acute and chronic pancreatitis in the context of mitochondrial disease is lacking. We decided to conduct a systematic literature review to summarise the clinical features, underlying genetics, treatment, and outcomes in published cases of acute and chronic pancreatitis in patients with a genetically confirmed mitochondrial disease.

## 2 Methods

A search was conducted on PubMed, SCOPUS and Web of Science (January 1993 to June 2024) to identify all published cases where a reported acute or chronic pancreatitis was present in association with genetically defined mitochondrial disease (search terms: mitochondrial disease, MELAS, MERRF, PEO, progressive external ophthalmoplegia, KSS, Kearns‐Sayre syndrome, Pearson syndrome, mtDNA mutation, mtDNA depletion, mtDNA deletion, POLG, acute pancreatitis, chronic pancreatitis and pancre*). To be included in the study, the publications had to be in English and report original patient data. In addition, they had to have a PubMed abstract, and the text needed to be available for review in paper or electronic format. Publications not fitting the criteria were excluded. The PRISMA guidelines were followed in the conduction and reporting of this study [13]. For a systematic review based on freely accessible public data, no ethical permission was required.

## 3 Results

**FIGURE 1:**
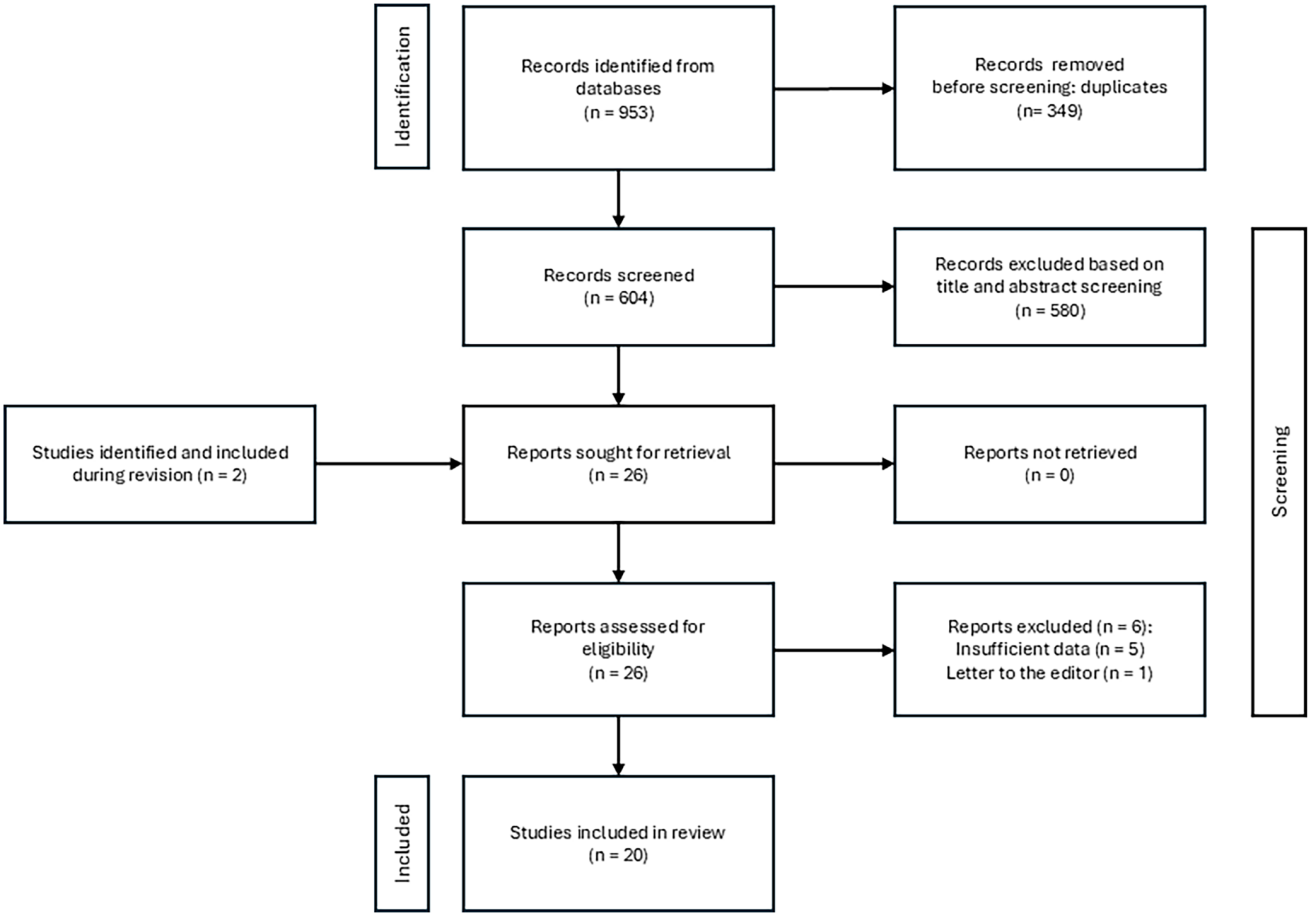
Flowchart of article screening process as per the PRISMA guidelines.

The literature search yielded 604 publications, of which 19 fit the inclusion criteria. During the manuscript revision process, two additional publications were identified and, after evaluation, one was included. Figure 1 shows the flowchart of the identification process of the studies that were selected for review.

The included publications reported a total of 24 individuals with genetically confirmed mitochondrial disease and a history of acute or chronic pancreatitis. The core features of the patients described in the identified studies are shown in Table 1. Among the 24 patients, 12 were women and 12 were men. Mean age at first recorded pancreatitis was 13 years and median age 10 years; age range was 3 months to 53 years.

**TABLE 1:** Mitochondrial disease patients with pancreatitis reported in the literature.

Patient	Original publication	Age at the time of first pancreatitis	Sex	Pancreatitis symptoms	Disease time course	Key laboratory findings	Genetic variant	Mitochondrial clinical syndrome
P 1	[14]	16 mo	M	N/A	Acute	Elevated amylase	m.3243A>G (heteroplasmy level > 95% in blood)	MELAS
P 2	[15]	10 yo	M	Recurrent vomiting lasting multiple days.	Recurrent/chronic	Amylase 256–602 U/L	m.3243A>G (heteroplasmy level 36%–76%^b^)	MELAS
P 3	[16]	27 yo	F	Asymptomatic	Recurrent/chronic	N/A	m.3243A>G	MIDD
P 4	[17]	22 yo	M	Asymptomatic	Recurrent/chronic	N/A	m.3243A>G (heteroplasmy level 10% in blood, 60% in urinary epithelium, 75% in muscle)	Other/Not specified
P 5	[17]	53 yo	F	Abdominal pain	Acute^a^	Lipase 1052 U/L	m.3243A>G (heteroplasmy level 20% in blood, 35% in urinary epithelium, 45% in muscle)	Other/Not specified
P 6	[17]	13 yo	M	Abdominal pain	Recurrent/chronic^a^	Elevated serum amylase and lipase	m.3243A>G (heteroplasmy level 15% in blood)	Other/Not specified
P 7	[18]	23 yo	F	Abdominal distension and vomiting	Acute^a^	Amylase 1158 U/L	m.3243A>G	MELAS
P 8	[19]	10 yo	M	N/A	Acute	Amylase 1616 U/L Lipase 4640 U/L	Large mtDNA deletion (m.7628_11297del)	Other/Not specified
P 9	[20]	13 mo	F	Persistent abdominal pain and vomiting.	Recurrent/chronic^a^	Lipase 1639–2908 U/L	Large mtDNA deletion	Pearson syndrome
P 10	[21]	N/A	M	N/A	Recurrent/chronic	N/A	Large mtDNA deletion (m.11521_14425del, heteroplasmy level 72% in blood)	Pearson syndrome
P 11	[21]	18 mo	F	N/A	Recurrent/chronic	N/A	Large mtDNA deletion (m.6141_9908del, heteroplasmy level 76% in blood)	Pearson syndrome
P 12	[22]	3 yo	M	One episode of abdominal pain, vomiting and lethargy of unknown aetiology at 2 yo.	Recurrent/chronic	Normal amylase and lipase	Large mtDNA deletion (m.6323_13706del)	Other/Not specified
P 13	[23]	15 yo	M	Abdominal pain, vomiting	Recurrent/chronic^a^	Amylase 264 U/L Lipase 609–3307 U/L	Large mtDNA deletion (4.6kbp)	KSS
P 14	[24]	16 mo	F	Abdominal pain and distension, mild fever, vomiting	Recurrent/chronic^a^	Lipase 3901–11 784 U/L	Large mtDNA deletion (m.7950_13993del)	Pearson syndrome
P 15	[25]	6 yo	F	Abdominal pain	Recurrent/chronic^a^	Amylase 1200 U/L Lipase 600 U/L	m.8344A>G	MERRF
P 16	[18]	4 yo	F	Abdominal pain and vomiting	Recurrent/chronic^a^	Amylase 830 U/L Lipase 1650 U/L	m.8344A>G	CPEO
P 17	[26]	11 yo	F	Tachycardia, paleness, peripheral coldness, diarrhoea, abdominal bloating	Recurrent/chronic^a^	Amylase 223–1924 U/L Lipase 820–5816 U/L	m.8993A>G	Leigh syndrome
P 18	[27]	8 yo	F	Abdominal pain, diarrhoea and vomiting	Acute^a^	N/A	c.2243G>C, c.2542G>A variants in *POLG*	Alpers syndrome
P 19	[28]	N/A	F	Abdominal pain, diarrhoea	Recurrent/chronic	N/A	c.713A>G, c.1303C>T variants in *RMND1*	Other/Not specified
P 20	[28]	N/A	M	Abdominal pain, diarrhoea	Recurrent/chronic	N/A	c.613G>T, c.713A>G variants in *RMND1*	Other/Not specified
P 21	[29]	17 yo	M	N/A	Acute	N/A	Neuropathological findings characteristic for Alpers syndrome	Alpers syndrome
P 22	[30]	N/A	M	N/A	Acute	N/A	‘MELAS syndrome diagnosed in childhood’, variant not specified	MELAS
P 23	[31]	27 yo	F	Abdominal pain, vomiting	Acute	Amylase 143 U/L Lipase 373 U/L	‘Genetically confirmed MELAS’, variant not specified	MELAS
P 24	[32]	3 mo	M	N/A	Acute	Lipase 441–548 U/L	Severe mtDNA depletion in skeletal muscle and liver	Other/Not specified

Abbreviations: CPEO, chronic progressive external ophthalmoplegia; KSS, Kearns–Sayre syndrome; MELAS, mitochondrial encephalomyopathy, lactic acidosis and stroke‐like episodes; MERRF, myoclonic epilepsy with ragged red fibres; MIDD, maternally inherited diabetes and deafness; mo, months old; mtDNA, mitochondrial DNA; yo, years old.

^a^ Diagnosis of acute pancreatitis fulfilling the Revised Atlanta Classification criteria, based on available data.

^b^ DNA extracted from peripheral blood, urinary sediment and hair follicle; tissue specific heteroplasmy levels not specified.

Nine of the individuals (38%) presented with acute pancreatitis with no previous history of pancreatitis episodes (P1, P5, P7, P8, P18 and P21–24). However, in a slight majority of cases (*n* = 15, 63%) pancreatitis was recurrent or chronic (P2–4, P6, P9, P10–17, P19 and P20). The most common symptoms were abdominal pain (*n* = 11, 46%) (P5, P6, P9, P13–16, P18–20 and P23) and vomiting (*n* = 8, 33%) (P2, P7, P9, P13, P14, P16, P18 and P23). Other occurring symptoms were abdominal distension (*n* = 3) (P7, P14 and P17), diarrhoea (*n* = 4) (P17–20) and mild fever (*n* = 1) (P14). One of the chronic pancreatitis patients (P3, Table 1) had no pancreatic symptoms in their medical history but showed signs of chronic pancreatitis in imaging. A 3‐year‐old boy (P12) with a large mtDNA deletion had an episode of abdominal pain, vomiting and lethargy with no indications of pancreatic involvement, but a year later presented asymptomatic with signs of chronic pancreatitis in abdominal ultrasound and MRCP. Ten case reports provided sufficient information to state that the patient fulfilled the Revised Atlanta classification for acute pancreatitis. In the remaining case reports, information on laboratory and reference values, symptoms or imaging findings was insufficient. These cases are classified as reported pancreatitis.

In 14 of the reported 24 patients, data on laboratory findings were provided. Elevated amylase was reported in 10 (71%) out of these 14 patients, while elevated lipase was reported in 11 patients (79%) (P5, P6, P8, P9, P13–17, P23 and P24). Both amylase and lipase were elevated in seven patients (50%) (P6, P8, P13, P15–17 and P23). The previously mentioned three‐year‐old boy with asymptomatic chronic pancreatitis (P12) had normal levels of amylase and lipase. For a 15‐year‐old patient [23] triglyceride concentrations were reported as slightly elevated (triglyceride 1.8 mmol/L; reference < 1.3 mmol/L). For other patients, triglyceride concentration data were not available.

Some form of imaging was reported as a diagnostic tool in 54% (*n* = 13) of the patient cases. Abdominal computed tomography (CT) was utilised in eight cases (P2, P5–7, P13 and P15–17) and abdominal ultrasound in four (P3, P12, P18 and P23). Magnetic resonance cholangiopancreatography (MRCP) was performed in four cases (P3, P12, P16 and P17) and endoscopic resonance cholangiopancreatography (ERCP) in two (P6 and P15). In one case report (P9), the used imaging method was not specified. Signs of acute or chronic pancreatitis were found in seven out of eight (88%) abdominal CTs performed. One abdominal CT taken at first presentation of acute pancreatitis was reported normal. However, CT imaging was repeated as abdominal pain persisted, revealing a stricture in the superior mesenteric artery, suggesting an ischemic cause of the acute pancreatitis (P5). Abdominal ultrasound findings indicated acute or chronic pancreatitis in three of the four (75%) cases. One abdominal ultrasound of a 27‐year‐old woman with the mitochondrial encephalomyopathy, lactic acidosis and stroke‐like episodes syndrome (MELAS, OMIM: 540000) and symptoms and laboratory findings indicative of pancreatitis was reported normal (P23). The results of all the performed MRCP and ERCP studies were unremarkable, proving intact pancreaticobiliary systems with no structural abnormalities. A cyst in the pancreatic tail with otherwise normal appearance of the pancreas was reported as a non‐specific imaging finding in a 23‐month‐old girl with Pearson syndrome and recurrent pancreatitis (P9).

Among the reported 24 patients with mitochondrial disease and a history of pancreatitis, the most common genetic aetiologies were large‐scale mtDNA deletions and the m.3243A>G variant in mtDNA (*n* = 7 for both). Among those with the m.3243A>G, three were reported to have the MELAS syndrome (P1, P2 and P7), and one the maternally inherited diabetes and deafness (MIDD, OMIM: 520000) phenotype (P3). In three patients with the m.3243A>G, no clinical syndrome was specified (P4–6). Among the patients with mtDNA deletion, four presented with the Pearson syndrome (P9–11 and P14), and one was reported with the Kearns–Sayre syndrome (KSS, OMIM: 530000) (P13). For two patients with mtDNA deletion, no syndromic phenotype was reported (P8 and P12). Among the three patients harbouring the m.8344A>G variant, one was reported presenting clinically with the myoclonic epilepsy with ragged red fibres (MERRF, OMIM: 545000) syndrome (P15), another with chronic progressive external ophthalmoplegia (CPEO, OMIM: 530000) (P16) and the third with Leigh syndrome (OMIM: 500017) (P17). In addition to mtDNA variants resulting in mitochondrial disease, one patient with compound heterozygous variants in *POLG* and Alpers' syndrome (OMIM: 203700) was reported (P18), as well as two patients with variants in *RMND1* (P19 and P20). In addition, there was one case of mtDNA depletion where a conclusive genetic diagnosis was not reached (P24). In the remaining three reported patients, a genetic diagnosis of mitochondrial disease was not reported. These patients were clinically diagnosed as MELAS (P22 and P23) and Alpers' syndrome (P21). Information on mtDNA variant heteroplasmy levels was provided in 11 case reports (P1, P2, P4–6, P9‐11, P15, P16 and P22). Numerical values were reported on seven patients, the highest reported heteroplasmy levels ranging from 72% to over 95%. For the remaining four cases, no numerical data on heteroplasmy levels were given. We also identified a paper reporting patients with mtDNA depletion syndrome associated with pathogenic variants in *MPV17*, where one patient with a history of pancreatitis was reported but no further details were given [33].

Medical therapy for pancreatitis was reported in eight of the 24 cases. A high dose of coenzyme Q10 was acutely administered to a 24‐year‐old man with MELAS and recurrent pancreatitis since age 10, with subsequently reported improvement of pancreatic symptoms (P2). A 10‐year‐old girl with recurrent pancreatitis associated with the MERRF syndrome since age 6 was started on coenzyme Q10, vitamins B1 and B2, and phenytoin, with more rare complaints of abdominal pain. No recurrence of pancreatitis during the follow‐up was reported (P15). Another 10‐year‐old girl with recurrent pancreatitis associated with CPEO since age 4 (P16) was started on coenzyme Q10 at a dose of 2 mg/kg/d, vitamin B1 at a dose of 6 mg/kg/d, and camostat mesylate with no further episodes of pancreatitis in the following 18 months. A 28‐year‐old woman with MELAS (P7) had a history of recurrent episodes of vomiting since age 16, which resolved in 2–3 days without treatment. She was admitted for abdominal distension and bile‐stained vomiting at the age of 23, and fasting therapy, gabexate mesylate, and sodium bicarbonate were started as treatment for pancreatitis. The next day, the patient's condition deteriorated suddenly with shock and acute respiratory distress syndrome leading to ICU admission. The medications were not continued after discharge, but the patient had since been kept on a low‐fat diet resulting in no reported episodes of pancreatitis or recurrent vomiting in the following five years. There were no mentions of the use of ketogenic diet in any of the reviewed publications.

Four patients underwent surgical procedures related to pancreatitis. A 54‐year‐old man with the m.3243A>G variant (P4) underwent cephalic pancreatectomy due to chronic pancreatitis. A 57‐year‐old woman with the m.3243A>G variant (P5) had surgery for a stricture in the superior mesenteric artery, which was suspected to have caused the pancreatitis. A 32‐year‐old man with the m.3243A>G (P6) variant underwent pancreatic sphincterotomy at age 17 as treatment for recurrent pancreatitis, but it was proven ineffective. Finally, a 13‐year‐old girl with Leigh syndrome and recurrent pancreatitis (P17) had a duodenal feeding tube inserted to prevent recurrence; however, the pancreatitis episodes persisted.

Eight of the patients were deceased at the time of the case report. The deaths of six patients happened shortly after being admitted to hospital with pancreatitis symptoms and laboratory/imaging findings, although not all case reports explicitly stated the cause of death. A 16‐month‐old boy with MELAS (P1) who was admitted for acute pancreatitis and hyperglycaemia died unexpectedly the day after admission due to cardiorespiratory collapse following acute gastrointestinal haemorrhage. A 3‐year‐old girl (P14) with Pearson syndrome and recurrent pancreatitis was admitted for another pancreatitis episode. Her clinical status deteriorated 16 days after admission, leading to ventilator support and death at age 3 years 4 months. A 13‐year‐old girl with Leigh syndrome (P17) had recurrent episodes of pancreatitis at ages 11–13 years resulting in a total of 13 hospital admissions. Recurrent pancreatitis persisted despite treatment, and she was switched to total parenteral nutrition and palliative care. She died at age 13. An eight‐year‐old girl (P18) with no history of pancreatitis was admitted for abdominal pain, diarrhoea and vomiting in addition to ultrasound findings indicative of acute pancreatitis. Her condition rapidly declined on the third day after admission, presenting with circulatory and respiratory insufficiency, jaundice and haemorrhaging diathesis. She died on the fifth day after admission due to hepatic failure. Alpers' syndrome was diagnosed *post mortem*. A 17‐year‐old boy (P21) had a progressive 8‐month‐long clinical course of severe headaches, multiple stroke‐like episodes with visual deficits and seizures concluding in acute haemorrhaging pancreatitis. He was diagnosed *post mortem* with Alpers' syndrome. A three‐month‐old boy (P24) was admitted for hypotonia, decreased urine output, hepatomegaly and generalised weakness with elevated lipase levels indicating pancreatitis. His condition rapidly declined, and he died at age 3 months 2 weeks from hepatic failure and deficiency of clotting factors causing massive gastrointestinal haemorrhage. A 54‐year‐old man (P4) with the m.3243A>G variant and asymptomatic chronic calcific pancreatitis died at age 54 due to a possible stroke‐like episode. A 23‐month‐old girl with Pearson syndrome and a history of recurrent pancreatitis (P9) developed transfusion‐dependent anaemia, progressive liver failure with cholestasis and severe intestinal dysfunction requiring total parenteral nutrition at age 20 months. She died at the age of 23 months from septic shock with multiple organ failure.

## 4 Discussion

The objective of this systematic review was to summarise underlying genetics, treatment and outcomes in published cases of acute and chronic pancreatitis in patients with a genetically confirmed mitochondrial disease. We conducted a search on three databases to ensure relevant case reports were identified as broadly as possible. The PRISMA guidelines were followed throughout this study [13].

The findings suggest that pancreatitis associated with mitochondrial diseases may occur at a younger age than in the general population. A recent retrospective study showed a median age of 65 in hospitalised acute pancreatitis cases, with ages ranging from 18 to 97 [34]. A similar report on paediatric patients in the United Kingdom aged 0–15 reported a median age of 11 at diagnosis of acute pancreatitis [35]. Conversely, in our patients aged 0–15, the median age at pancreatitis was 5 years, while for those patients above the age of 18, the median age was 27 years.

In another recent review, it was noted that there is increasing evidence of mitochondrial dysfunction itself being a key event in the pathophysiology of acute pancreatitis [36]. The detailed mechanism in pancreatitis in mitochondrial disorders remains unclear, although it may be related to increased endoplasmic reticulum stress and metabolic defects within pancreatic cells, leading to decreased exocrine enzyme secretion, which in turn could result in intracellular protease accumulation and activation [26]. Our findings fall in line with this, as the patients didn't present with classical pancreatitis aetiologies, nor did they have structural abnormalities in the pancreaticobiliary systems in imaging, apart from a single documented anomaly of a stricture in the superior mesenteric artery.

Mortality of our reviewed patients was relatively high. Eight out of the 24 patients had died at the time of the case report, with the deaths of six patients (25%) shortly after admission to hospital with pancreatitis‐related symptoms and findings. However, it should be noted that the causes of death were not explicitly stated in all case reports. Akca et al. reported an in‐hospital mortality rate of 1.8% in Turkish acute pancreatitis patients aged 18–97 [34], while paediatric pancreatitis patients from the Global Burden of Disease database showed a mortality rate of 0.04 deaths per 100 000 children [37].

One paper reported nine patients with a genetic diagnosis of KSS or Pearson syndrome who had pancreatitis in their medical history [38]. However, very little clinical information on the individual patients was provided. Despite this weakness in data granularity, this paper further supports the association between mitochondrial diseases and pancreatitis.

Limitations of this systematic review include a small number of patients and heterogeneity in original patient case reporting. It is also possible that some cases of pancreatitis in association with mitochondrial disease that have been reported remained undetected by the literature searches used in this systematic review. We could not confirm whether all patients fulfilled the Revised Atlanta Classification for pancreatitis, as some original publications did not provide enough information on the disease course, including symptoms, imaging findings and specific laboratory tests and reference values. In addition, some case reports only mentioned pancreatitis as part of the medical history of the patients. In many cases, the mtDNA variant heteroplasmy levels were not given in the original papers. However, the original papers clearly indicate that these were individuals with clinically manifest mitochondrial disease, not asymptomatic variant carriers.

Medical therapy in individual cases was in part experimental. Of importance, the reported cases where symptom improvement was observed in association with coenzyme Q10 or vitamin B supplementation are not proof of a beneficial effect of these treatments. The current data do not enable us to suggest specific treatment rationales for pancreatitis in the context of mitochondrial disease. Comprehensive information on genetic studies performed on the patients was also scarce within the case reports. Furthermore, genetic testing has taken huge leaps of improvement since the 1990s, thus the earliest case reports in this review lack the full spectrum of knowledge we have on mitochondrial disease genetics today.

The method of systematic review limits our ability to conclusively address the question of pancreatitis aetiology. However, since there are several reports of pancreatitis in patients with mitochondrial disease where no conventional risk factors have been detected, we suggest it is probable that at least some forms of mitochondrial disease are associated with an elevated risk of pancreatitis. A systematic summary of published cases provides a more comprehensive outlook on the existing data and allows for some systematic patterns to emerge.

Pancreatitis is a relatively rare but relevant potential complication of mitochondrial disease. Pancreatitis seems to be associated particularly with the m.3243A>G variant and large‐scale mtDNA deletions, and first presentation is commonly in childhood or young adult age. Typical risk factors and imaging findings associated with pancreatitis in the general population are absent. We conclude that this potentially life‐threatening condition merits increased attention.

## Author Contributions

**Olivia Hahl:** data collection and initial data analysis, writing the first draft, manuscript revision. **Mika H. Martikainen (guarantor author):** original research plan, overview of the study, data analysis, manuscript revision.

## Funding

The authors have nothing to report.

## Ethics Statement

The authors have nothing to report.

## Conflicts of Interest

The authors declare no conflicts of interest.

## Data Availability

The data that support the findings of this study are available from the corresponding author upon reasonable request. 1. Have you in the past five years accepted the following from an organisation that may in any way gain or lose financially from the results of your study or the conclusions of your review, editorial or letter.
